# Endoplasmic Reticulum Stress Stimulates p53 Expression through NF-κB Activation

**DOI:** 10.1371/journal.pone.0039120

**Published:** 2012-07-30

**Authors:** Wan-Chi Lin, Yu-Chi Chuang, Yung-Sheng Chang, Ming-Derg Lai, Yen-Ni Teng, Ih-Jen Su, Clay C. C. Wang, Kuan-Han Lee, Jui-Hsiang Hung

**Affiliations:** 1 Eppley Institute for Research in Cancer and Allied Diseases, University of Nebraska Medical Center, Omaha, Nebraska, United States of America; 2 Department of Biochemistry and Molecular Biology, College of Medicine, National Cheng Kung University, Tainan, Taiwan; 3 Institute of Basic Medicine, College of Medicine, National Cheng Kung University, Tainan, Taiwan; 4 Infectious Diseases and Signaling Research Center, College of Medicine, National Cheng Kung University, Tainan, Taiwan; 5 Department of Biological Sciences and Technology, National University of Tainan, Tainan, Taiwan; 6 National Institute of Infectious Diseases and Vaccinology, National Health Research Institutes, Tainan, Taiwan; 7 Department of Pharmacology and Pharmaceutical Sciences, School of Pharmacy, University of Southern California, Los Angeles, California, United States of America; 8 Institute of Pharmaceutical Science, Chia Nan University of Pharmacy and Science, Tainan, Taiwan; 9 Department of Biotechnology, Chia Nan University of Pharmacy and Science, Tainan, Taiwan; University of Hong Kong, Hong Kong

## Abstract

**Background:**

Induction of apoptosis by endoplasmic reticulum (ER) stress is implicated as the major factor in the development of multiple diseases. ER stress also appears to be a potentially useful major response to many chemotherapeutic drugs and environmental chemical compounds. A previous study has indicated that one major apoptotic regulator, p53, is significantly increased in response to ER stress, and participates in ER stress-induced apoptosis. However, the regulators of p53 expression during ER stress are still not fully understood.

**Principal Findings:**

In this report, we demonstrate that induction of p53 expression is mediated through NF-κB signaling pathways during ER stress in MCF-7 cells. Tunicamycin or brefeldin A, two ER stress inducers, increased p53 expression in MCF-7 and Hela cells. We found p53 nuclear localization, activity, and phosphorylation at serine 15 on p53 increased during ER stress. Nuclear translocation of NF-κB and activity of NF-κB were also observed during ER stress. ER stress-induced p53 expression was significantly inhibited by coincubation with the NF-κB inhibitor, Bay 11-7082 and downregulation of NF-κB p65 expression. The role of p53 in mediating Brefeldin A-induced apoptosis was also investigated. Induction of p53 expression by Brefeldin A was correlated to Brefeldin A-induced apoptosis. Furthermore, downregulation of p53 expression by p53 siRNA significantly reduced Brefeldin A-induced apoptosis in MCF-7 cells.

**Significance:**

Taken together, NF-κB activation and induction of p53 expression is essential for ER stress-induced cell death which is important for therapeutic effects of clinical cancer drugs. Our results may provide insight into the mechanism of cancer chemotherapy efficacy that is associated with induction of ER stress.

## Introduction

In eukaryotic cells, the endoplasmic reticulum (ER) is a dynamic membranous organelle which plays an important role in protein folding, transport, and processing. Many chemical agents, viral proteins, and adverse metabolic conditions cause protein misfolding or protein accumulation in the ER, leading to ER stress. Research over the past decade has also demonstrated that many physiological conditions cause ER stress, e.g. nutrient or glucose deprivation, degenerative neuronal disorders [1], [2], type II diabetes [3], [4], differentiation of B-cells into plasma cells [5], [6], and virus infection [7]–[9]. In this study, tunicamycin and brefeldin A were used to induce ER stress and activate complex signaling pathways known as the unfolding protein response (UPR) and the ER-overloading response pathway (EOR) [10], [11].

UPR pathway has three components in mammalian cells: basic leucine zipper transcription factor ATF6, IRE1 RNA-processing enzyme, and ER localized kinase (PERK). Previous studies have indicated that activation of NF-κB is through calcium release, reactive oxygen species production, IRE1, and PERK signal pathway during ER stress [12], [13], [14], [15], [16]. We have also characterized the NF-κB response and found that NF-κB was activated through multiple pathways, including calcium signaling and pp38 kinase [17]. Activation of NF-κB is known to regulate expression in more than 100 genes, which are involved in diverse cell processes, such as cell proliferation, differentiation, apoptosis, and inflammation and immune responses [18].

Severe or prolonged ER stress induces activation of unique pathways that lead to cell death through apoptosis. Recently, several pathways have been directly implicated in ER stress-induced apoptosis, including the caspase-12/caspase-4, CHOP/GADD153, IRE1/PERK/JNK, and p53 signaling pathways [19], [20], [21], [22]. p53 tumor suppressor is a nuclear protein that functions as a regulator of transcription and mediates several biological effects, such as growth arrest, senescence, and apoptosis in response to various forms of stress [23]. Elevation of p53 expression during ER stress in MEFs, MCF-7 and HCT116 cell lines has been reported [22]. p53 has been demonstrated to play an important role in the dysregulation of ER [24], [25]. Although elevation of p53 gene expression during ER stress has been described, it has been unclear whether and how p53 gene expression is regulated in response to ER stress. In addition, UPR has been shown to regulate glycogen synthase kinase (GSK) 3β which is essential for the regulation of p53 and cyclin D1 degradation during early ER stress [26], [27], and p53 protein was downregulated at 3–6 h with tunicamycin or brefeldin A treatment. Because ER dysfunction has been linked to many diseases, it is important to investigate the mechanism by which p53 expression is regulated during ER stress.

It is interesting to note that NF-κB plays a role in p53 expression in certain situations [28]. NF-κB may specifically recognize an NF-κB site on the p53 promoter and activates the p53 promoter [29]. Because ER stress activates NF-κB, we hypothesized that ER stress induces p53 expression may through NF-κB activation. In this report, we demonstrate ER stress induced by tunicamycin and brefeldin A leads to increased expression of p53 and that the increased expression of p53 is mediated by NF-κB. Furthermore, when we knocked down p53 expression by p53 RNAi, the cells were more resistant to tunicamycin and Brefeldin A treatment. Therefore, in these ER stress conditions, induction of p53 is required to induce cell death. Taken together, expression of p53 in response to ER stress is regulated by NF-κB, and participates in ER stress-induced cell death. The NF-κB appears to play dual roles in regulating cell survival and death under ER stress.

## Materials and Methods

### Chemicals

Tunicamycin, Brefeldin A, ethidium bromide, diethyl pyrocarbonate, actinomycin D and SDS were obtained from Sigma. Bay 11-7082 and cycloheximide were purchased from Calbiochem. ECL Western blot detection system was from Amersham Biosciences. The RT-PCR reagent, proTaq plus, oligo(dT)_15_ primer, M-MLV-Reverse transcriptase, dNTP, and G418 were purchased from Promega (Madison, WI). The NE-PER nuclear and cytoplasmic extraction reagents kit and Micro BCATM protein assay reagent kit were from Pierce. Anti-GRP78 was purchased from Transduction Laboratories. The anti-p53 was purchased from Oncogene (Merck Ltd., Budapest, Hungary). The following antibodies from Cell Signaling (Beverly, MA) were used: anti-p-Ser^15^-p53, anti-p-Ser^20^-p53, anti-p-Ser^37^-p53, and anti-p-Ser^46^-p53 antibodies. Anti-IκBα, anti-Bax, anti-MDM2, anti-p65, and anti-NF-κB p50 antibodies were obtained from Santa Cruz Biotechnology (Santa Cruz, CA). Anti-γ-tubulin was from MDBio (Frederick, MD). Topoisomerase II was from NEOMAKERS. Anti-β-actin was from Chemicon (Pittsburgh, PA). LipofectAMINE 2000, Dulbecco's modified Eagle's medium (DMEM), and antibiotic mixture (10,000 units of penicillin, 10,000 mg of streptomycin) and trypsin-EDTA were products of Invitrogen. Fetal bovine serum was obtained from Biological Industries (Beit Haemek, Israel).

### Cell Culture

MCF-7 and Hela cell lines were obtained from ATCC (Manasses, VA, USA). MCF-7 pSuper stable transfectant, and MCF-7 pSuper p53 stable transfectant transfectant cell lines were generated form MCF-7 and HCT116, and these cell lines were maintained at 37°C in a 5% CO_2_ atmosphere in DMEM supplemented with 10% heat-inactivated fetal bovine serum, 2 mM L-glutamine, 100 units/ml penicillin, and 100 µg/ml streptomycin.

### Plasmid and Stable Clone Cell Lines Construction

pSuper and pSuper p53 siRNA plasmid were provided gratis from Brummelkamp. The original pSUPER vector is the is the H1 RNA polymerase III promoter, which drives the endogenous production of shRNA. is the H1 RNA polymerase III promoter, which drives the endogenous production of shRNA. is the H1 RNA polymerase III promoter, which drives the endogenous production of shRNA. H1 RNA polymerase-III promoter to direct intracellular synthesis of siRNA like transcripts. The two pLKO.1-shRNA vectors used for knockdown of NF-κB p65 are the following: TRCN0000014684 (shRelA) and TRCN000014686 (shRelA). The two shRelA vectors were obtained from National RNAi Core Facility (Taipei, Taiwan). MCF-7 cells were co-transfected with pCDNA3.1/ pSuper or pcDNA3.1/pSuper p53 plasmids by using Invitrogen LipofectAMINE 2000 reagent according to the manufacturer's protocol. Cells were then selected by G418 for 2 weeks. The pCDNA3.1/ pSuper and pcDNA3.1/pSuper p53 stable clone cell lines were established by western blotting.

### Preparation of Cytosolic and Nuclear Extracts

Subcellular fractionation was performed using NE-PER Nuclear and Cytoplasmic Extraction Reagents kit according to the manufacturer's instructions. In brief, MCF-7 (1×10^6^) cells in 10-cm dishes were exposed to 1 µg/ml Brefeldin A in 10% FBS–containing DMEM for 0, 6, 12, and 24 h. After treatment, the cells were washed with cold calcium and magnesium free PBS, collected with a cell scraper, and harvested by centrifugation. The cell pellets were suspended in 200 µL of Cytoplasmic Extraction Reagent I solution and incubated on ice for 10 min, followed by adding 11 µL of Cytoplasmic Extraction Reagent II solution and incubation on ice for 1 min. The cell suspensions were centrifuged at 16,000×*g* for 5 min to collect the supernatant cytoplasmic fraction. The pellets were resuspended with 100 µL of Nuclear Extraction Reagent on ice for 40 min. This suspension was centrifuged at 16,000×*g* for 10 min at 4°C to collect the supernatant nuclear fraction.

### Western immunoblotting

Cells, seeded in 10-cm dishes (1×10^6^ cells per dish), were incubated for 16 h, subjected to different drug treatments, and harvested by scraping. Cell lysates were prepared by treating cells with 2X SDS lysis buffer (0.1 M Tris (pH 6.8), 0.4% SDS, and 20% glycerol). The protein concentration of the supernatant was measured using a Micro BCATM protein assay reagent kit. To the cell lysate, the same volume of SDS-PAGE sample loading buffer [100 mmol/L Tris-HCl, 4% SDS, 5% β-mercaptoethanol, 20% glycerol, and 0.1% bromophenol blue (pH 6.8)] was added, and the cells were boiled for 10 min. Equal amounts of proteins were resolved in SDS-polyacrylamide gels and transferred onto polyvinylidene fluoride membranes (Pierce). Following blocking with 5% nonfat dry milk for 1 h at room temperature and washing with Tween 20 with Tris-buffered saline (TTBS), the polyvinylidene fluoride membranes were incubated overnight at 4°C with primary antibody in TTBS containing 1% bovine serum albumin. The second anti-mouse antibody-horseradish peroxidase conjugate (1∶2000 dilutions) was subsequently incubated with membranes for 1 h at room temperature and washed extensively for 40–50 min with TTBS at room temperature. The blots were probed with the ECL Western blot detection system according to the manufacturer's instructions.

### RT-PCR and real-time PCR analysis of p53 mRNA

After treatment, the cells were washed with cold PBS and then cells were harvested. Total RNA was extracted from MCF-7 cells using TRIzol reagent (Invitrogen) and chloroform extraction. RT was performed using 2 μg of total RNA. cDNA synthesis was performed using 200 U of Moloney murine leukemia virus reverse transcriptase, 5 μM oligoDT, 1 mM dNTP solution, and 3 mM Mg^2+^ in a volume of 20 μl. The PCR reaction was performed using the following primers:

*p53* gene: (F) 5′-GAGGTTGGCTCTGACTGTACC-3′/(R) 5′-CCTCATTCAGCTCTCGGAA C-3′. *GRP78* gene: (F) 5′-TAGCGTATGGTGCTGCTGTC-3′/(R) 5′-GTCAGGCGATTCTG GTCATT-3′. *GAPDH* gene: (F) 5′-CCATCAATGACCCCTTCAT-3′/(R) 5′-TGGACTGTGT CATGAGTCC-3′. The products were visualized after electrophoresis on a 1.5% agarose gel containing ethidium bromide. The signal level of the bands was quantified densitometrically. In real-time PCR, PCR was performed for the resulting RT products using oligonucleotide primers specific for p53, and GAPDH. The primers used were as follows: *p53* gene (F) 5′-GCG CACAGAGGAAGAGAATC-3′/(R) 5′-CCTCATTCAGCTCTCTCGGAAC-3′; *GAPDH* gene (F) 5′-GATTCCACCCATGGCAAATTC-3′/(R) 5′-AGCATCGCCCCACTT GATT-3′. All PCR reactions were performed with ABI 7500 Fast Real-Time PCR System using DNA-binding SYBR Green dye for detection of the PCR products and results were analyzed by ABI StepOne Software version 2 (Applied Biosystems).

### Chromatin immunoprecipitation assay (CHIP)

MCF-7 cells were plated into 100 mm-diameter dishes for overnight incubation and stimulated with or without 1 µg/ml of Brefeldin A for 0, 3, 9, and 24 h. The CHIP assay was performed according to the supplier's directions (Millipore-Upstate, Billerica, MA). Briefly, treated cells were fixed in serum-free medium containing 1% formaldehyde for 10 minutes at 37°C. MCF-7 cells were cross-linked with 1% formaldehyde for 10 min. The cells were then harvested, lysate, and sonicated to shear genomic DNA into fragments of 200–1000 base pairs. An aliquot was also retained as an input sample to normalize PCR reactions and analyze shearing efficiency. 400 μg of each remaining chromatin preparation was incubated overnight at 4°C with 2 µg of anti-NF-κB p50, anti-NF-κB p65, and anti-p53 antibodies. Both immunoprecipitated and input DNA samples were analyzed by PCR to determine the relative amounts of DNA from the *p53* gene promoter region present in the samples. The primers used for amplification by PCR were: (F) 5′CAC CAG GTC GGC GAG AAT CCT3′/(R) 5′CTC TAG ACT TTT GAG AAG CT3′.

The PCR products were visualized after electrophoresis on a 1.5% agarose gel containing ethidium bromide.

### Electrophoretic mobility shift assay (EMSA)

EMSA were conducted using LightShift chemiluminescent EMSA kits (Pierce). Briefly, nuclear extracts (5 μg total protein) were incubated with approximately 15 pmol of biotinylated oligonucleotides probe corresponding to the NF-kB consensus sequences in p53 promoter (5′-TGGGATTGGGGTTTTCCCCTCC-3′, containing the kB site from the p53 promoter) in 25 mmol l^–1^ Hepes (pH 7.6), 100 mmol l^–1^ NaCl, 15 % glycerol, 0.1 % NP-40 and 0.5 mmol l^–1^ PMSF in a final volume of 20 μl. Incubations were conducted for 60 min at room temperature. After incubation, mixtures were applied to 5 % acrylamide non-denaturing gels and electrophoresed for approximately 2 h at 100 V. Gels were then transferred to nylon membranes *via* electroblotting at 380 mA for 30 min. NF-κB–p53 probe complexes on each blot were visualized by a chemiluminescent reaction with streptavidin/horseradish peroxidase, according to the manufacturer's protocol, and visualized with the BioSpectrum AC imaging system (UVP, CA) according to the manufacturer's instructions.

### Immunofluorescence

MCF-7 cells were seeded at 2×10^5^ per well in 6-well flat-bottomed plates and incubated in 10% FBS–supplemented DMEM for 24 h. Cells were treated with 2.5 μg/ml tunicamycin or 1 μg/ml Brefeldin A in the same medium. Cells for immunofluorescence microscopy of NF-κB and p53 were fixed with 3.7% paraformaldehyde for 10 min and washed three times with PBS. Cells were then treated with ice-cold methanol for 2 min and washed three times with PBS. Cells were stained for NF-κB and p53 translocation using anti-p65 and p53 antibodies overnight at 4°C and then anti-mouse FITC-conjugated antibody for 1 h. After staining with antibody, cells were viewed with a fluorescence microscope.

### Cell viability Assay

Cell viability was assessed with a 3-(4,5-dimethylthiazol-2-yl)-2,5-diphenyltetrazolium- bromide (MTT) assay in three replicates. MCF-7 and stable transfectant cells were seeded at 3×10^5^ per well in 24-well flat-bottomed plates and incubated in 10% FBS–supplemented DMEM for 24 h. Cells were treated with 5 μg/ml Tunicamycin and 1 µg/ml BFA in the same medium. Controls received DMSO vehicle at a concentration equal to that in drug-treated cells. After 48 h, the drug-containing medium was replaced with 200 µL of 10% FBS–supplemented DMEM containing 0.5 mg/mL MTT, and cells were incubated in the CO_2_ incubator at 37°C for 4 h. Medium was removed, the reduced MTT was solubilized in 600 µL per well of DMSO, and 100 µL aliquots from each well was transferred to 96-well plates to measure absorbance at 570 nm.

### Luciferase reporter assay

The p53 and NF-κB transcriptional activity was performed by luciferase reporter assay. MCF-7 cells were transfected with pp53-TA-Luc (p53 response element) or NF-κB-responsed promoter plasmid (NF-κB response element) by using Invitrogen LipofectAMINE 2000. After 16 h incubation, the cells were treated with or without Tunicamycin or Brefeldin A at the time indicated. The luciferase activity was measured using a Promega luciferase assay system according to the manufacturer's protocol. The firefly luciferase activity was normalized to that of Renilla luciferase.

### Statistical analysis

Results were presented as the mean ± S.D., and statistical comparisons were made using the Student's *t* test. Significance was defined at the *p*<0.05 or 0.01 levels.

## Results

### Intense and prolonged ER stress induces p53 protein expression

**Figure 1:**
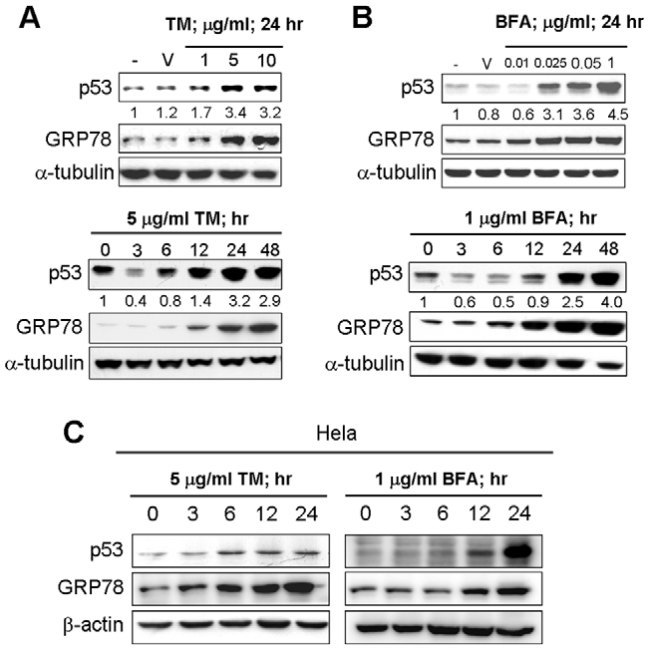
p53 expression is elevated in response to endoplasmic reticulum stress. (A) and (B) present the dose- and time- dependent effect of ER stress inducer, tunicamycin and brefeldin A, on p53 expression. MCF-7 Cells were exposed to tunicamycin and brefeldin A in 10% FBS–supplemented DMEM as time and dose indicated. The cell lysates were analyzed by western blotting with antibodies for p53, GRP78, and α-tubulin. The p53 protein expression level was quantified densitometrically. GRP78 served as an ER stress marker. (C) Induction of p53 expression by ER stress in three different cell lines. Hela cells were treated with 5 µg/ml tunicamycin in 10% FBS–supplemented DMEM as time indicated. The cell lysates were analyzed by western blotting with specific antibodies for p53 and β-actin.

Many studies have indicated that p53 tumor suppressor is one of the central players in the response of cells to various forms of stress. MCF-7 human breast adenocarcinoma cells were treated with tunicamycin or brefeldin A, and no changes in p53 protein level in the control cells were observed (Fig. 1A, 1B, upper panel). In contrast, when MCF-7 cells were incubated with tunicamycin or brefeldin A, an increase in the level of p53 was observed in a dose and time-dependent manner. In a dose-dependent manner, p53 protein started to increase in 1 µg/ml of tunicamycin and 0.025 µg/ml of brefeldin A (Fig. 1A, 1B, upper panel). In a time-dependent manner, the level of p53 protein was induced at 12–48 h after treatment with tunicamycin (Fig. 1A lower panel) and the p53 level at 24 hr was approximately 3-fold higher than that expressed at 0 hr. Similarly, using another ER stress inducer, brefeldin A, p53 protein was significantly induced with time up to 48 hr. For example, the p53 level at 48 hr was enhanced up to about 4-fold compared with 0 hr (Fig. 1B Lower panel). Expression of GRP78 protein was used to check that treatments in our system indeed resulted in ER stress. Notably, we observed that under the tunicamycin or brefeldin A treatment, p53 protein was first downregulated at the early time course point of 3 and 6 hr before its expression was increased. This finding is consistent with previous studies showing that mild ER stress increased cytoplasmic localization and degradation of endogenous p53 in human primary WI-38 cells and HT1080 cells [30] and led to downregulation of p53 protein in human lung adenocarcinoma A549 cells [26]. Furthermore, we demonstrated that upregulation of p53 expression by ER stress is not restricted to a particular cell line. In the human cervical cancer Hela cells, treatment of tunicamycin or brefeldin A also induced p53 expression to the extent as seen in the MCF-7 cells (Fig. 1C). All these results suggested that increase of p53 protein expression level by ER stress is a general phenomenon that can occur in a variety of human cells regardless of stimulus types that initiate this process.

### Induction of p53 is transcription-dependent

**Figure 2:**
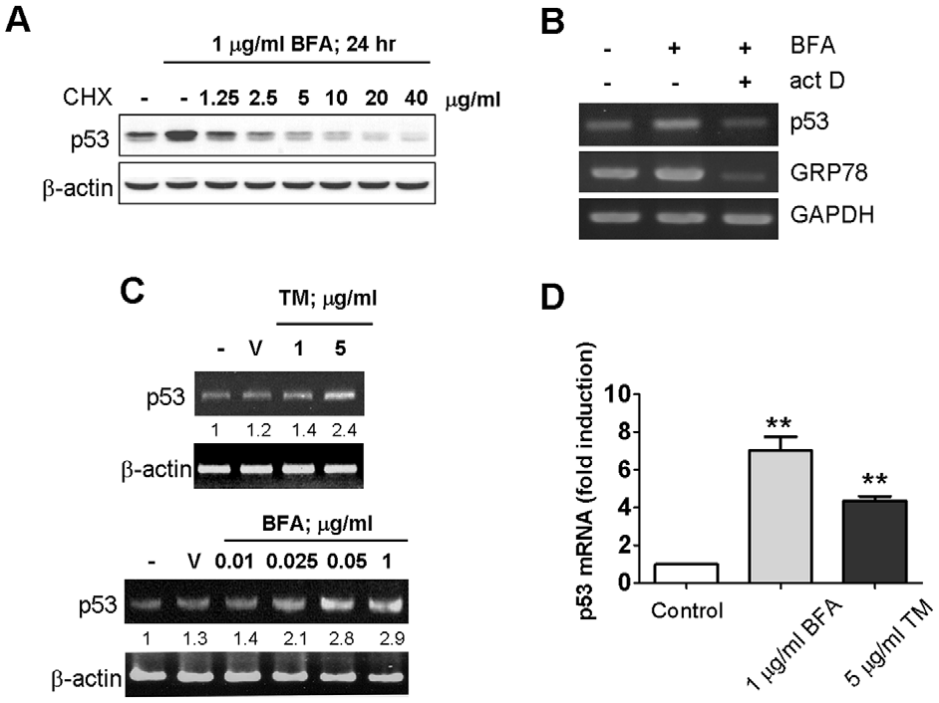
Induction of p53 expression is regulated at the transcriptional level during ER stress. (A) Induction of p53 expression by BFA was inhibited by protein synthesis inhibitor cycloheximide in MCF-7 cells. The cells were exposed with 1 μg/ml brefeldin A in the presence of the various concentrations of cycloheximide for 24 hr. The expression of p53 and β-actin were analyzed by Western blotting. (B) The p53 mRNA expression was inhibited by actinomycin D during ER stress. MCF-7 cells were treated 1 μg/ml brefeldin A with or without 20 μg/ml actinomycin D for 24 hr and harvested for total RNA isolation. The mRNA level of p53, GRP78 and GAPDH were determined by RT-PCR. (C) The effect of ER stress on p53 mRNA expression, with MCF-7 cells treated with 1 µg/ml brefeldin A in the dose- and time-dependent manner. The total RNA was isolated and then subjected to RT-PCR analysis. The p53 mRNA expression level was quantified densitometrically. (D) The level of p53 mRNA in response to ER stress was determined by real-time RT-PCR. MCF-7 cells were treated with 1µg/ml brefeldin A or 5 µg/ml tunicamycin for 24 hr and harvested for total RNA isolation. Real-time RT–PCR was performed as described in Materials and methods. *Columns,* mean of three independent experiments; bars, SD (**, *P*<0.01, Student's t test).

To establish the mechanism of p53 expression by ER stress, protein biosynthesis and transcription inhibitors were used in brefeldin A-induced p53 expression in MFC-7 cells. A treatment of a low dose of cycloheximide (1.25 µg/ml) was sufficient to reduce ER stress-activated p53 protein expression, almost 100% to its basal level (Fig. 2A). In addition, we also used transcriptional inhibitor, actinomycin D, to examine whether the event of induction of p53 is transcription-dependent. MFC-7 cells were co-treatment of actinomycin D with brefeldin A and, we used reverse transcription-PCR to analyze the expression levels of p53 mRNA. The p53 mRNA levels were dramatically decreased by actinomycin D in response to ER stress (Fig. 2B). Furthermore, by the analyses of conventional and real-time RT-PCR, we observed that p53 expression was already increased at an mRNA level when MCF-7 cells were treated with tunicamycin or brefeldin A (Fig. 2C, 2D). These data together suggested that the elevated expression of p53 protein during ER stress, at least partially, is contributed by increasing the expression of p53 transcripts through a regulation on p53 promoter activity.

### ER stress enhances nuclear localization and phosphorylation of p53, as well as p53-mediated transcriptional activation

**Figure 3:**
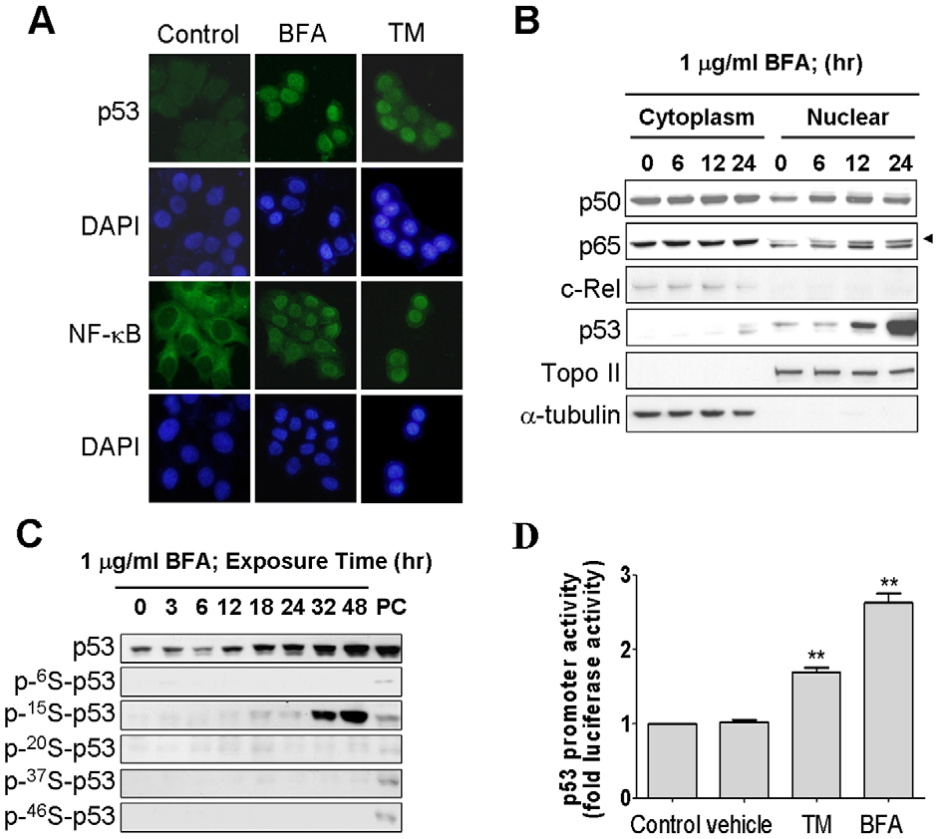
Increased p53 phosphorylation and activity by ER stress. (A) Nuclear localization of NF-κB and p53 in response to ER stress in MCF-7 cells. The cells were treated with 1 μg/ml BFA, and the localization of NF-κB and p53 were determined by using immunofluorescence staining. (B) NF-κB and p53 nuclear localization were observed in a time-dependent manner after 0, 6, 12, and 24 h exposure to BFA in MCF-7 cells. The cells were treated with 1 μg/ml brefeldin A as time indicated, and NF-κB subunits in the cytoplasmic and nuclear fractions were analyzed by Western blotting with antibodies against NF-κB subunits p65, p50, c-Rel, and p53. Topoisomerase II and α-tubulin were used as internal markers for nuclear and cytoplasmic proteins. (C) time-dependent effect of BFA on p53 phosphorylation. MCF-7 cells were treated with 1 μg/ml brefeldin A in 10% FBS–supplemented DMEM as time indicated. The total cell lysates subjected to immunoblotting with antibodies against anti-p53, anti-p-ser^6^-p53, anti-p-ser^15^-p53, anti-p-ser^20^-p53, anti-p-ser^37^-p53, and anti-p-ser^46^-p53. Exposure of MCF-7 cells to 50 J/m2 of UV light served as positive control. (D) ER stress induced transcriptional up-regulation of p53. MCF-7 cells were transfected with reporter vectors encoding firefly luciferase driven by p53 promoter, pp53-TA-Luc plasmid. Transfectants were treated with tunicamycin or Brefeldin A for 24 h. Luciferase activities were normalized to that of cotransfected Renilla luciferase. Columns, mean of three independent experiments; bars, SD (*, *P*<0.05, Student's t test).

The main function of p53 protein is work as a transcription factor that regulates its target genes to mediate cellular activities. Nuclear localization and phosphorylation of p53 on its N-terminal transactivation domain are both essential for p53 function [31], [32]. Therefore, we next investigated the distribution of p53 in MCF-7 cells following the treatment of ER stress inducers. As indicated in Fig. 3A, very little p53 was detected in the untreated cells; however, p53 signal was significantly enhanced and presented exclusively in nuclei in the tunicamycin or brefeldin A-treated cells. To validate this microscopically visualized p53 localization, total cell lysates collected from MCF-7 cells were fractionated to separate cytoplasmic and nuclear components, and a western blotting analysis was performed to measure p53 protein level in each subcellular compartment. The majority of p53 protein was found to reside in nuclei. A sharp accumulation of p53 in the nuclear fractions was detected when cells were treated with brefeldin A for 12 and 24 hours (Fig. 3B). This result agreed with the immunostaining data shown in Fig. 3A, exhibiting a clear nuclear localization of p53 induced by ER stress. Subsequently, we examined phosphorylation status of p53 to see whether any serine residue on the N-terminus of p53 is phosphorylated when ER stress was induced. To this end, MCF-7 cells were treated with brefeldin A, and phosphorylation of p53 was analyzed by western blotting using p53 phospho-specific antibodies. Based on the fact that UV light can induce p53 phosphorylation at multiple sites including ser6, ser15, ser20, ser37, and ser46 [31], [32], MCF-7 cells exposed to 50 J/m2 of UV light were used to serve as a positive control for this experiment. As shown in the Fig. 3C, phosphorylation of p53 was observed at ser15, one of the most important p53 phosphorylation sites responsible for p53 transactivation function, thereby suggesting that ER stress-induced p53 protein is functional and is very likely transcriptionally active. To test transactivation ability of p53 protein, MCF-7 cells were transfected with a p53 reporter vector encoding a luciferase reporter gene under the control of a p53-responsive element. In the luciferase assay, tunicamycin or brefeldin A treatment both were shown to activate the expression of luciferase reporter (Fig. 3D), demonstrating that ER stress-induced p53 protein is transcriptionally active. Higher p53-depedent transcriptional activity is induced in the cells that undergo ER stress.

### Induction of p53 expression by ER stress is coupled with NF-κB activation

**Figure 4:**
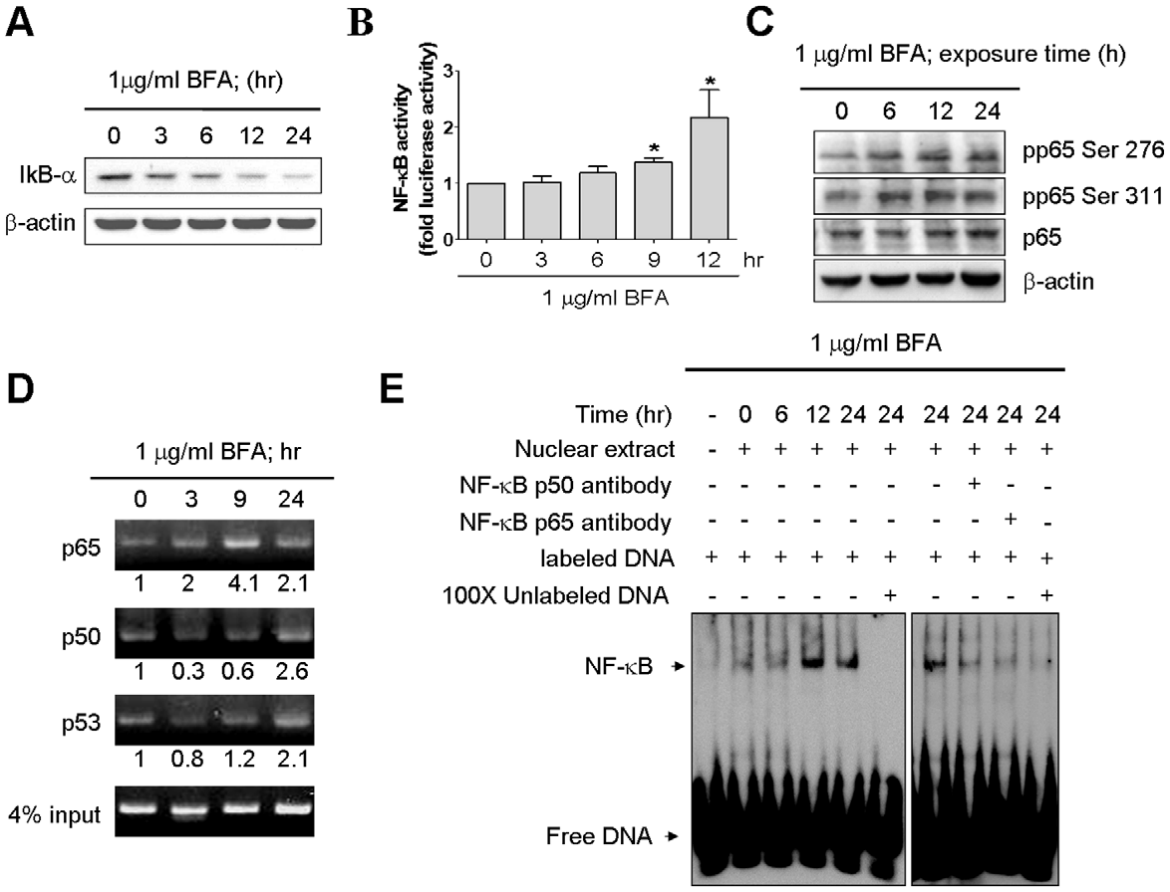
Induction of NF-κB activity during ER stress. (A) The effect of BFA on IκBα expression in MCF-7 cells. The cells were incubated with 1 µg/ml Brefeldin A in 10% FBS–supplemented DMEM for 0, 3, 6, 12, and 24 h. The total cell lysates were analyzed by Western blotting with antibodies for IκBα and β-actin. (B) The effect of 1 µg/ml brefeldin A on NF-κB phosphorylation in MCF-7 cells. The cells were incubated with 1 µg/ml BFA as time indicated. The whole cell lysates were subjected to immunoblotting with antibodies against anti-p65, anti-p-ser^276^-p65, anti-p-ser^311^-p65 and β-actin. (C) Analysis of NF-κB activity during ER stress in MCF-7 cells. The cells were transfected with reporter vectors encoding firefly luciferase driven by NF-κB-induced promoter, and transfectants were treated with BFA as time indicated. Luciferase activities were normalized to that of cotransfected Renilla luciferase. Columns, mean of three independent experiments; bars, SD (*, *P*<0.05, Student's t test). (D) Nuclear NF-κB DNA binding activity was measured by EMSA using a probe corresponding to the NF-κB-binding site of the p53 promoter. The *arrows* indicated the NF-κB complexes, and the supershifted band was induced with anti-p65 subunit or anti-NF-κB p50 subunit antibody.(E) ER stress induced NF-κB subunits p50, p65, and p53 binding to p53 gene promoter in MCF-7 cells. MCF-7 cells were treated with 1 µg/ml BFA for for 0, 3, 9, and 24 h. Nuclear lysates were generated and immunoprecipitated with anti-NF-κB p50, anti-NF-κB p65, and anti-p53 antibodies. Immunoprecipitated DNA was anayzed for p53 promoter by PCR and gel electrophoresis. 4% of input served as loading control.

NF-κB family members have been previously suggested to have a role to regulate p53 gene in response to certain types of stress [33]. Based on these findings, we hypothesized that activation of NF-κB may be the mechanism that accounts for the increased expression of p53 during ER stress. To examine this hypothesis, we performed western blotting to measure protein level of IκB-α, an inhibitor protein of NF-κB, which functions to sequester NF-κB in the cytoplasm. A marked decrease of IκBα was detected in the MCF-7 cells treated with brefeldin A (Fig. 4A), and the corresponding increase in the nuclear localization of NF-κB (p50 and p65) was also been seen in the treated cells within a similar time frame (Fig. 3A, 3B). The phosphorylation of NF-κB (p65) at serine 276 and serine 311 has been previously suggested to be important for NF-κB transcriptional activation [34], [35]. We therefore examined the phosphorylation status of p65 using antibodies specifically against phosph-Ser276 or phosph-Ser311 on p65 protein. By the analyses of western blotting, we observed that upon brefeldin A stimulation, the phosphorylation levels of p65 at serine 276 and serine 311 were enhanced (Fig. 4B), suggestive of the functionality of p65 that acts as a transcription factor during the process of ER stress. In addition, we also performed a luciferase reporter assay to further assess the ability of activated NF-κB to drive target gene expression. For this purpose, we transfected MCF-7 cells with a NF-κB reporter plasmid that was constructed by numerous copies of consensus NF-κB-binding sequence and a luciferase reporter gene, and then challenged these transfectants with brefeldin A. Utilizing a luciferase assay, we demonstrated that NF-κB transcriptional activity was significantly increased in the MCF-7 cells following the brefeldin A treatment (Fig. 4C). Furthermore, we performed an electrophoretic mobility shift assay (EMSA) to analyze binding activity of NF-κB on *p53* promoter using the nuclear extracts isolated from MCF-7 cells and a labeled double-stranded probe that bears the consensus NF-κB-binding sequence of *p53* promoter. As shown in the left panel of Fig. 4D, NF-κB and DNA binding activity was markedly increased, particularly at the time point of 12 and 24 hour post-treatment. The upper gel-shifted band was further depleted by anti-NF-κB p50 and p65 antibodies to the binding reactions. The disappearance of NF-κB/DNA complex revealed that two NF-κB family members p50 and p65 were involved (Fig. 4D, right panel). To further validate that interaction between NF-κB proteins and *p53* promoter indeed occur *in vivo*, we conducted a chromatin immunoprecipitation (CHIP) assay. Cross-linked chromatin was isolated from MCF-7 cells, immunoprecipitated by anti-p65 or anti-p50 antibodies and then used as a template in a quantitative PCR to amplify a small DNA fragment targeted to the *p53* promoter. Consistent with the EMSA result, data from the CHIP assay showed that treatment of brefeldin A for 24 hours enhanced the association of both p65 and p50 proteins with *p53* promoter (Fig. 4E), providing the direct evidence to support the role of NF-κB in modulation of *p53* gene expression during ER stress.

### NF-κB activity is required for the increased expression of p53 in response to ER stress

**Figure 5:**
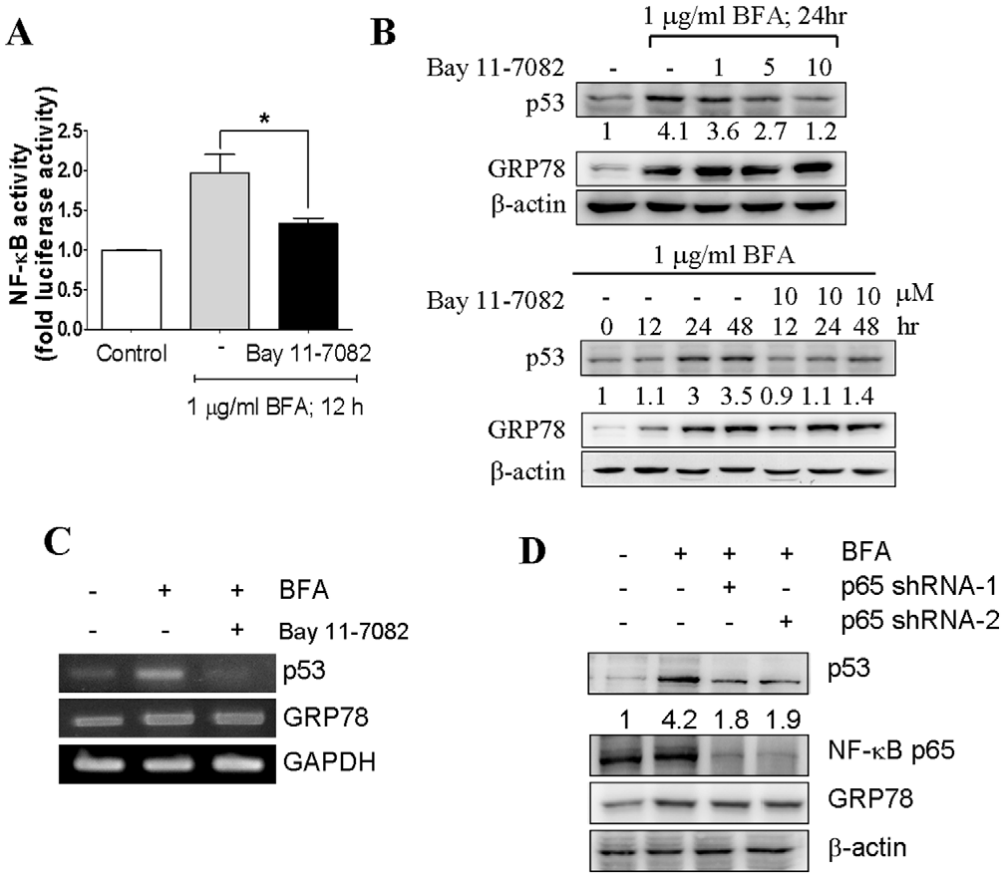
Regulation of p53 expression depends on NF-κB signaling pathway in ER stress. The effect of the pharmacologic inhibitor of NF-κB, Bay 11-7082, on NF-κB activity. (A) The cells were transfected with NF-κB activation reporter and the transfectants were treated 1 µg/ml Brefeldin A with or without 10 µM Bay 11-7082 for 12 h. Luciferase activities were normalized to that of cotransfected Renilla luciferase. Columns, mean of three independent experiments; bars, SD (*, *P*<0.05, Student's t test). (B) Bay 11-7082 inhibits the effect of ER stress-activated p53 expression in MCF-7 cells. Cultures of MCF-7 cells were treated with 1 µg/ml Brefeldin A in the presence of Bay 11-7082 in the dose- and time-dependent manner. The cell lysates were analyzed by immunoblotting with p53, GRP78, and β-actin antibodies. (C) The effect of Brefeldin A or combination with NF-κB inhibitor on p53 mRNA expression in MCF-7 cell line. Total RNA was isolated from MCF-7 cells and then subjected to RT-PCR analysis with p53, GRP78 and G3APDH specific primers. (D) Downregulation of NF-κB p65 by shRNA decreased p53 protein level during ER stress. Cells were transfected with NF-κB p65 shRNA plasmid, and the whole cell lysates were subjected to immunoblotting with antibodies against anti-p53, anti-p65, anti-GRP78 and β-actin.

To test whether attenuation of NF-κB signaling affects the increased expression of p53 protein, we used an inhibitor Bay11-7028 attempting to inhibit NF-κB activity. By an assessment using a NF-κB reporter assay, we showed that the concentration of the NF-κB inhibitor applied in this experiment was able to efficiently reduce NF-κB activity that was activated by brefeldin A (Fig. 5A). To further investigate the effect of Bay11-7082 on ER stress-induced p53 expression, co-treatment of Bay11-7028 with brefeldin A dramatically decreased the induction of p53 at both protein and mRNA levels (Fig. 5B, 5C). To more specifically inhibit NF-κB signaling, expression of the p65 subunit of NF-κB was knocked down by two p65 shRNAs targeting different p65 sequences respectively. MCF-7 cells were transfected with p65-shRNA plasmids and measured the brefeldin A-triggered p53 induction in these p65 knock-down cells. We observed that the increased expression of p53 was abolished in the p65-shRNA transfected cells, proving that NF-κB signaling is required for ER stress-induced p53 expression (Fig. 5D).

### GSK-3β does not involve in p53 expression that is induced by the prolonged ER stress

**Figure 6:**
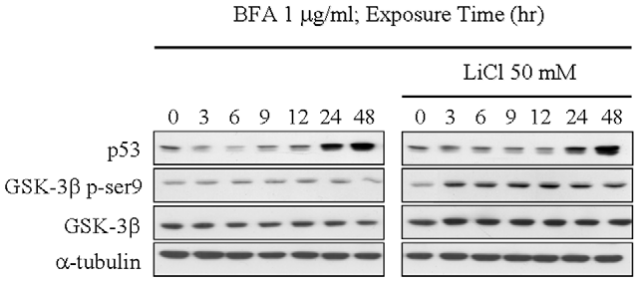
GSK3β signaling pathway does not involve in p53 expression through ER stress. Effects of GSK-3β inhibitor, lithium chloride, on p53 expression in ER stress. MCF-7 cells were incubated 1 µg/ml Brefeldin A with or without lithium chloride as time indicated. The total lysates were subjected to immunoblotting with antibodies against anti-p53, anti-GSK-3β, p-ser^9^-GSK-3β, and α-tubulin.

Previous studies have indicated that when mild ER stress is induced, p53 protein abundance is regulated through the GSK-3β signaling transduction pathway [26], [30]. To investigate whether GSK-3β is also involved in regulation of p53 expression if cells are challenged with more prolonged or intense ER stress, we incubated MCF-7 cells with brefeldin A in the absence or presence of GSK-3β inhibitor, lithium chloride (LiCl), for a time as indicated. Application of LiCl in our experiment was shown to successfully block GSK-3β activity by increasing phosphorylation of GSK-3β at ser9. However, even when GSK-3β is inactivated, induction of p53 expression was still observed (Fig. 6). Therefore, this result showed that GSK-3β does not involve in regulation of p53 expression in response to the prolonged ER stress.

### Induction of p53 is required for ER stress-induced cell death

**Figure 7:**
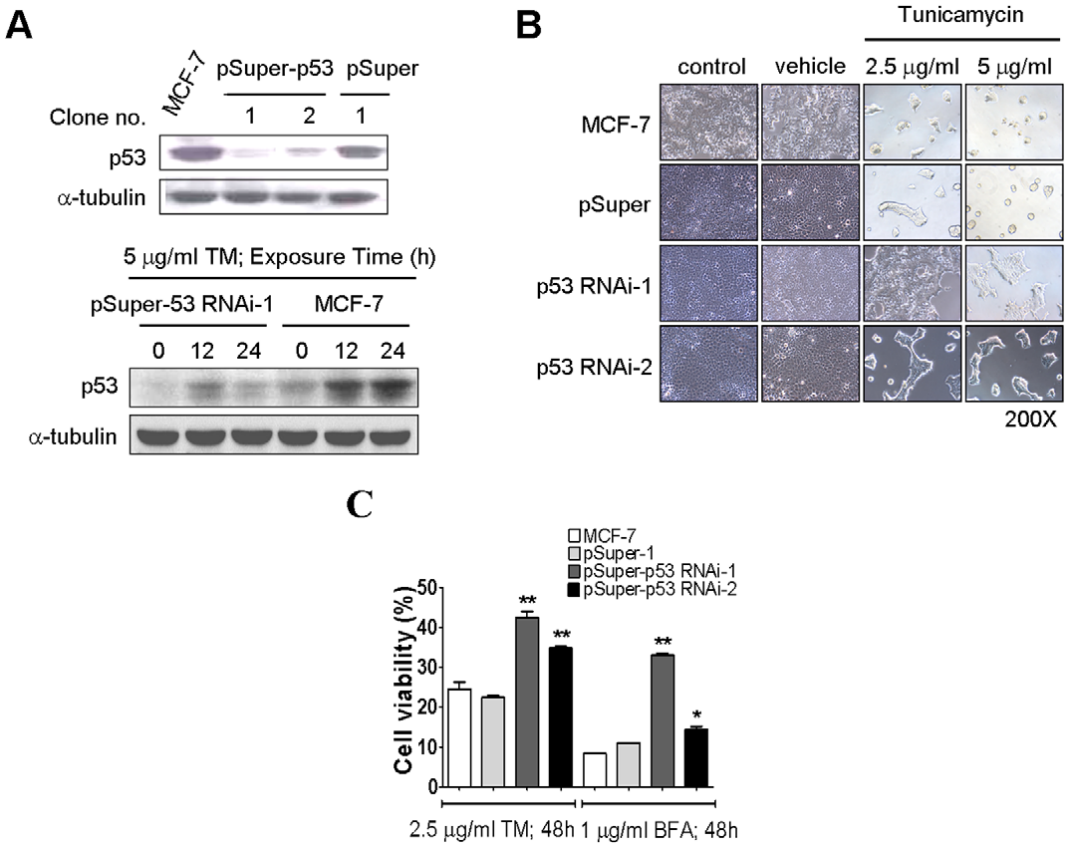
Knockdown of p53 protects cells against BFA-induced cell death. Down-regulation of p53 expression by p53 RNAi during ER stress. (A) MCF-7 cells were cotransfect with pcDNA3.1/pSuper or pcDNA3.1/pSuper-p53 RNAi plasmid by Lipofectammine 2000. The stable transfectants were selected by G418, and then expression of p53 was analyzed by immunoblotting (upper panel). The cell growth rate of MCF-7, MCF-7-pSuper, MCF-7-pSuper-p53-1, and MCF-7-pSuper-p53-2 cells were performed by MTT assay (lower panel). Furthermore, western blot analyses of the expression levels of p53 in MCF-7 cells were subjected to tunicamycin treatment (lower panel). (B) The morphologic changes after a 48-hour 2.5 µg/ml and 5 µg/ml tunicamycin treatment of MCF-7 cells. The cells were followed by photography under phase-contrast magnification. (C) shRNA-mediated knockdown of p53 protects MCF-7 cells from Tunicamycin- or Brefeldin A-induced cell death. The stable transfectants were incubated with Tunicamycin or Brefeldin A in 10% FBS–supplemented DMEM for 48 hr. Cell viability was measured by the MTT assay. *Columns,* mean of three independent experiments; bars, SD (*, *P*<0.05; **, *P*<0.01, Student's t test).

To address how important the induction of p53 is to the ER stress-induced cell death, we transfected pSuper-siRNA-p53 vector into MCF-7 cells to knockdown p53 expression. Two lines of stable transfectants were generated and named as pSuper-p53 RNAi-1 and pSuper-p53 RNAi-2. The efficiency of *p53* gene silencing was evaluated using western blotting. Transfectants pSuper-p53 RNAi-1 appeared to have a better knockdown effect than pSuper-p53 RNAi-2 (Fig. 7A, upper panel). Also, these transfectants, which expressed pSuper-siRNA-p53 construct, were shown to efficiently reduce both basal and ER stress-induced level of p53 protein (Fig. 7A, lower panel). To study the effect of p53 knockdown in ER stress, we compared the morphology changes between MCF-7 cells and their transfectants following tunicamycin treatment. In the MCF-7 and MCF-7 pSuper vector control cells, tunicamycin treatment resulted in a dose-dependent, progressive change in cell shape from flat to round, while in the transfectants pSuper-p53 RNAi-1 and pSuper-p53 RNAi-2, tunicamycin is more limited to cause this change (Fig. 7B). Finally, an MTT assay was applied to measure cell survival after ER stress was induced. Treatment of MCF-7 cells and MCF-7 pSuper vector control cells with tunicamycin or brefeldin A for 48 hours induced severe cell death. In contrast, knockdown of p53 prevented cells from tunicamycin or brefeldin A-triggered cell death (Fig. 7C), thereby indicating that p53 is indispensible in the process of ER stress-induced cell death.

## Discussion

**Figure 8:**
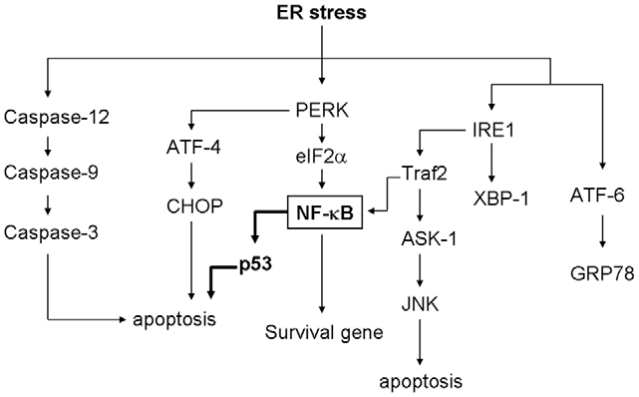
Signaling of p53 expression by NF-κB during endoplasmic reticulum stress. Accumulation of unfolded or misfolded proteins in the endoplasmic reticulum induces activation of multiple signaling pathways. Many studies have indicated that activation of IRE1, ATF6, and PERK represents the standard UPR pathways and turns on the expression of many downstream genes such as *GRP78*. ER stress induced caspase-12/caspase-9/caspase-3, PERK/ATF-4/CHOP, IRE1/Ask1/JNK, and PERK/eIF2α/NF-κB signaling pathways and then those pathways may alter cellular homeostasis by regulating multiple genes, resulting in regulation of cell death or survival. This pathway (indicated by *heavy arrows*) is demonstrated in this report. We demonstrated that induction of p53 expression is mediated by NF-κB during ER stress.

Prolonged ER stress leads to cell apoptosis. Several signal transduction pathways have been identified that can explain how cells trigger programmed cell death when faced with unfolded protein accumulation. ER stress can induce multiple signal pathway involving in ER stress-induced apoptosis, such as caspase12-caspase-9-caspase-3, PERk-ATF-4-CHOP, IRE-1-ASK1-JNK, and p53 pathways [19], [36], [37], [23]. Here, we have demonstrated that ER stress can induce the expression of p53, and the induction is dependent on the transcription factors NF-κB. Induction of p53 expression through NF-κB by ER stress plays an important role in ER stress-induce cell death (Fig. 8). In this report, the contribution, function and regulation of p53 in ER stress-induced apoptosis in MCF-7 cells has been investigated. Although ER stress induced expression of p53 and p53 target gene have been described, the regulation of p53 gene expression was still unclear. We demonstrate that expression of p53 is induced by ER stress, and the p53 induction is dependent on the transcription factors NF-κB. We also show that p53 phosphorylation and nuclear localization is induced in response to ER stress. NF-κB activation and phosphorylation are increased by ER stress. p53 is required for ER stress-induced cell death in MCF-7 cells as demonstrated by the results from the p53-targeted siRNA experiment. These results suggest that induction of p53 expression by ER stress participates in ER stress-induced cell death. In addition, NF-κB in regulation of p53 expression evinces pro-apoptosis effects in response to ER stress.

Wild-type p53 is inactivated through cytoplasmic sequestration in a subset of human tumor cells. The levels and localization of p53 are tightly regulated by several posttranslational mechanisms, such as protein stability, phosphorylation, and subcellular localization. Previous studies have demonstrated that phosphorylation of p53 at S315 and S376 is required for its nuclear export and degradation of p53 is mediated by Hdm2 within 3 h of ER stress treatment [26]. In our study we observed p53 degradation occurred after treating with tunicamycin or brefeldin A in the period of 3 to 6 h. After 12 h treatment of brefeldin A, however, p53 was significantly induced by ER stress. Based on this phenomenon, there appears to be degradation of p53 in the early period of ER stress, however, when adaptation fails, prolonged and excessive ER stress induces expression of genes encoding mediation of cell death, such as CHOP and p53. This represents a mechanism of last resort among multicellular organisms to dispense with dysfunctional cells.

Previously, UPR has shown that GSK3β is involved in the cytosolic localization of p53 during ER stress, which prevents p53-dependent apoptosis during early ER stress [26], [30]. In addition to p53, GSK3β regulates the activation of caspase-2 during ER stress in leukemia [38]. GSK3β is required for the induction of long-chain acyl-CoA synthetase 3 (ACSL3) and lipid accumulation in response to ER stress [39]. On the other hand, previous study also indicated that GSK3β has been shown to affect NF-κB and STAT family members in the inflammatory process [40], [41]. In addition, GSK3β also participates in regulation of CHOP expression during ER stress in neuronal cells [42]. Therefore, we tried to co-incubate brefeldin A and the GSK3β inhibitor, lithium chloride, with MCF-7 cells, and found lithium chloride did not affect p53 expression in response to ER stress. The result indicates that GSK3β signal pathway is not involved in induction of p53 expression during ER stress. When normal mammalian cells are subjected to stress signals (e.g. hypoxia, radiation, DNA damage or chemotherapeutic drug) p53 is activated. With regard to p53 modifications, phosphorylation has been studied most intensively and has been proposed to play a critical role in the stabilization and activation of the tumor suppressor. Multiple serine (6, 9, 15, 20, 33, 37, 46, 315, 371, 376, 378, and 392) and three threonine residues (18, 55, and 81) have been reported to undergo phosphorylation in response to diverse stresses. We therefore investigated the role of p53 phosphorylation on five key serine residues (Ser6, Ser15, Ser20, Ser37, and Ser46) for p53 activation. The result showed that phosphorylation of p53 at ser15 was induced in response to ER stress. Many studies have exhibited ser15 phosphorylation of p53 in cells exposed to ionizing radiation, UV irradiation and chemotherapy drugs [32], [43]. p53 phosphorylation on S15 and other sites has been linked to apoptosis by chemotherapeutic drugs and chemopreventive agents. For example, induction of p53 phosphorylation on ser15 is caused by Topo I and Topo II inhibitors in relation to ATM and Chk2 activation [44]. In vitro evidence suggests that DNA damage-induced p53 Ser15 phosphorylation can block the interaction of p53 with its inhibitor, MDM2, and stimulate association with transcriptional co-activator proteins such as p300 [31]. Phosphorylation of p53 on Ser 15 and Ser37 sites can be regulated by ATM, ATR and DNA-PK, thus inhibiting the ubiquitin degradation of p53, promoting p53 activation and accumulation [45]. Therefore, our finding of p53 phosphorylation on Ser15 by ER stress shows these processes may participate in the regulation of ER stress-induced cell death. However, it is unclear what role Ser15 phosphorylation plays in ER stress processes and how this modification affects context-dependent programs of p53-regulated gene expression and their biological consequences. The relationship between p53 phosphorylation on Ser15 site and ER stress-induced cell death warrants further investigation.

Previous study indicated that regulation of NF-κB signaling pathway plays an important role during ER stress. From our previous results, we demonstrated that induction of COX-2 expression was mediated through NF-κB and p38 pathways in response to ER stress [17]. The role of NF-κB in COX-2 expression may suggest that NF-κB plays anti-apoptosis effects in response to ER stress. In addition to NF-κB and COX-2, ER stress can enhance proinflammatory NF-κB activation via C/EBP Homologous Protein (CHOP) and maintain an increased level of IL-8 production in human intestinal epithelial cells [46]. There is modulation of cell death in heart failure by NF-κB, which NF-κB activation required for ER stress-mediated apoptosis, whereas abrogation of myocyte NF-κB shifted the ER stress response to one of adaptation and survival [47]. These results implicated, in response to ER stress, ER-initiated pathways signal alarm by activating NF-κB transcription factor that induces expression of genes encoding mediation of host defense. Activation of both the PERK and IRE1 pathways leads to regulation of the NF-κB-IKK signaling pathway during ER stress through activation of IKK or degradation of the p65 subunit. The ATF6 branch can also regulate NF-κB activity [48]. All of these signals contribute to the triggering of apoptotic responses when ER stress is excessive, prolonged, or insufficiently neutralized, and all UPR sensors are activated and lead to the induction of both pro-apoptotic and anti-apoptotic factors. Previous studies have shown that multiple pathways are involved in ER stress-induced apoptosis, such as caspase12, PERk-eIF2α, IRE-1-JNK, and p53 pathways. Persistent ER stress can also trigger a switch in the UPR signal pathways from prosurvival to proapoptoic pathways, such as PERk-eIF2α and IRE-1-JNK pathways. On the other hand, many studies have also indicated that three phases of adaptation, alarm, and apoptosis mechanisms are involved in regulating prolonged ER stress [49]. When adaptation fails, incessant ER stress can trigger the signal alarm by activation of NF-κB. In PERk-eIF2α pathway, PERK protein activates its intrinsic kinase activity, resulting in phosphorylation of eIF2α and induction of NF-κB activity. For IRE-1-TRAF2 pathway, Ire1 binds TRAF2, signaling downstream kinases activation of NF-κB and c-jun, causing expression of genes associated with alarm and proapoptotic phases. Based on those research findings, NF-κB appears to play an important role in regulating signal pathways associated with ER stress-induced cell survival and apoptosis. Because during ER stress, ER-initiated pathways signal alarm by activation of NF-κB a transcription factor that induces expression of genes encoding mediators of host defense such as COX-2. However, Excessive and prolonged ER stress, activation of NF-κB turns to regulate cell death-associated genes expression such as p53, resulting in increase cell apoptosis. In addition, previous study indicated that increased level of ER stress which sensitizes drugs-resistant cells, and combination of ER stress-inducing agents is a novel therapeutic strategy for cancer cells [50], [51]. Our data provides a molecular basis for consideration of employing ER stress in killing of cancer cells directly or sensitizing the cells to other cytotoxic chemotherapy.

In summary, ER stress causes cell death by increasing the expression of p53 and activating NF-κB which is involved in the enhanced expression of p53. Previous studies have indicated that chemotherapeutic drugs cause cell death through ER stress, such as with Cisplatin [52]. These results may provide an important therapeutic strategy for chemotherapeutic drugs through ER stress-associated signaling pathways.
